# Effective Resistivity in Collisionless Magnetic Reconnection

**DOI:** 10.1038/s41598-018-28851-7

**Published:** 2018-07-12

**Authors:** Z. W. Ma, T. Chen, H. W. Zhang, M. Y. Yu

**Affiliations:** Institute for Fusion Theory and Simulation, Department of Physics, Zhejiang University, Hangzhou, 310027 China

## Abstract

An effective resistivity relevant to collisionless magnetic reconnection (MR) in plasma is presented. It is based on the argument that pitch angle scattering of electrons in the small electron diffusion region around the X line can lead to an effective, resistivity in collisionless plasma. The effective resistivity so obtained is in the form of a power law of the local plasma and magnetic field parameters. Its validity is confirmed by direct collisionless particle-in-cell (PIC) simulation. The result agrees very well with the resistivity (obtained from available data) of a large number of environments susceptible to MR: from the intergalactic and interstellar to solar and terrestrial to laboratory fusion plasmas. The scaling law can readily be incorporated into existing collisional magnetohydrodynamic simulation codes to investigate collisionless MR, as well as serve as a guide to *ab initio* theoretical investigations of the collisionless MR process.

## Introduction

Magnetic reconnection (MR) often occurs in plasmas containing sheared magnetic fields and can efficiently convert magnetic energy into the kinetic and thermal energies of the charged particles^1–3^. The process plays important roles in the evolution of the solar corona^4,5^, the geomagnetic tail^6,7^, the magnetosphere^8,9^, the intergalactic and interstellar, as well as laboratory plasmas^10,11^. In particular, collisionless or fast MR (FMR) on time scales much less than the inter-particle collision time can occur. FMR has often been attributed to anomalous resistivity arising from local current-instability driven turbulence in the small electron diffusion region of the MR^12,13^. However, the evolution and effect of the turbulence during the MR are difficult to follow and remain unclear. Existing studies^14,15^ have noted that the lifetime of a particle in the diffusion region can be considered as an effective collision time, since in terms of its momentum and energy changes, the electron dynamics in the electron diffusion region resembles that of electrons being scattered by collisions, except that here the scattering partners are the local magnetic and electric fields. In particular, Speiser^14^ introduced an *ad hoc* resistivity (more precisely, conductivity) based on the behavior of the electric current flow in the diffusion region. However, the problem remains unclear and no general conclusion can be drawn^14–16^.

It is well known that pitch-angle scattering of electrons in highly bending magnetic fields such as that in the diffusion region around the X point of MR can lead to particle momentum transfer from the parallel to the perpendicular initial current direction. In this paper, we reconsider the dynamics of electrons in this small region. The transit times of typical electrons in the region near the X line are determined by following their motion as the FMR process evolves. An effective resistivity in the form of power-law scaling of the most relevant local plasma parameters is obtained by replacing the mean-free-time in the expression for the collisional resistivity by an ensemble averaged electron transit time that depends on the local plasma and field parameters in the diffusion region. Validity of our approach is confirmed by full particle-in-cell (PIC) simulation of the FMR. Moreover, when compared with a large number of plasmas susceptible to MR: from the intergalactic and interstellar space to solar and terrestrial, as well as fusion, plasmas, it is found that the effective resistivity agrees very well with that estimated from the known parameters of these plasmas. The scaling law can readily be incorporated into the existing macroscopic MHD simulation codes^17^ for investigating FMR in complex space and fusion plasmas, as well as serve as guide for detailed theoretical investigation of the FMR physics.

## Analytical formulation of effective resistivity

**Figure 1:**
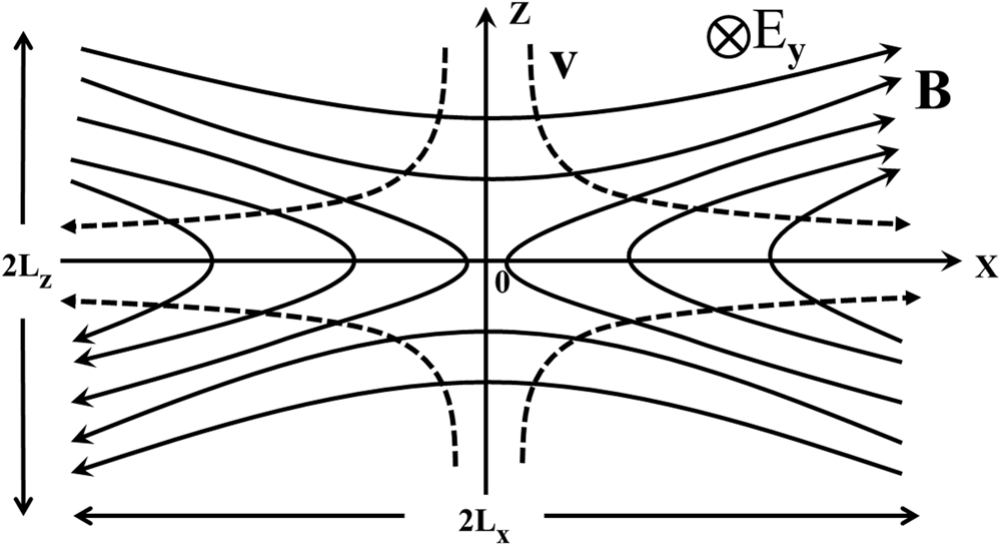
The local magnetic field ${\boldsymbol{B}}={{B}}_{x}z\hat{{\boldsymbol{x}}}/{L}_{z}+{{B}}_{z}x\hat{{\boldsymbol{z}}}/{L}_{x}$ and induction electric field ${\boldsymbol{E}}={{E}}_{y}\hat{y}$ in the diffusion region. The current sheet is in the *y* direction. The magnetic field increases (from null) with the distance from the X line, which is in the *y* direction and appears in the *x*, *z* plane here as the X point at (0, 0). Several idealized electron trajectories are shown as dashed curves.

Accordingly, collisionless MR can be investigated by replacing the mean free time *τ*_*mft*_ between collisions in the collisional resistivity *η*_*coll*_ = *m*_*e*_/*ne*^2^*τ*_*mft*_, where *n*, *e*, and *m*_*e*_ are the electron number density, charge, and mass, respectively, by the mean transit time ${\bar{\tau }}_{transit}$ of electrons in the diffusion region around the X line (see Fig. 1). The resulting effective, or collisionless, resistivity ${\eta }_{eff}={m}_{e}/n{e}^{2}{\bar{\tau }}_{transit}$ can then be implemented in the existing theories and MHD simulation codes. In the following, we shall obtain ${\bar{\tau }}_{transit}$ by concentrating only on, in our opinion, the most relevant physics involved.

The local magnetic and electric fields in the electron diffusion region of the MR (Fig. 1) can be approximated by

1 $${\boldsymbol{B}}={{B}}_{x}\frac{z\hat{{\boldsymbol{x}}}}{{L}_{z}}+{{B}}_{z}\frac{x\hat{{\boldsymbol{z}}}}{{L}_{x}}$$

2 $${\boldsymbol{E}}={{E}}_{y}\hat{{\boldsymbol{y}}}{\boldsymbol{,}}$$

where *L*_*x*_ and *L*_*z*_ are the characteristic lengths of the electron diffusion region, respectively, and the coefficients *B*_*x*_, *B*_*z*_(<<*B*_*x*_), and *E*_*y*_ are constants. When *L*_*z*_ << *L*_*x*_, the current sheet becomes elongated. This MR geometry is ofter referred to as of Y type.

The electron trajectory near the X line is then governed by

3 $${d}_{t}^{2}x=-e{v}_{y}{B}_{z}x/{L}_{x}{m}_{e}$$

4 $${d}_{t}^{2}z=e{v}_{y}{B}_{x}z/{L}_{z}{m}_{e}$$

where for simplicity we shall assume (consistent with the PIC simulation results below) that the electron velocity *v*_*y*_ in the current sheet varies only a little from the average value 〈*v*_*y*_〉 ~ −*J*_*y*_/*ne*.

Equations (3) and (4) yield

5 $$x(t)={x}_{0}\,\cosh (t/{\tau }_{x})+{v}_{x0}{\tau }_{x}\,\sinh (t/{\tau }_{x})$$

6 $$z(t)={z}_{0}\,\cos (t/{\tau }_{z})+{v}_{z0}{\tau }_{z}\,\sin (t/{\tau }_{z})$$

where ${\tau }_{x}=\sqrt{|{m}_{e}{L}_{x}/e{v}_{y}{B}_{z}|}$ and ${\tau }_{z}=\sqrt{|{m}_{e}{L}_{z}/e{v}_{y}{B}_{x}|}(\ll {\tau }_{x})$, and (*x*_0_, *z*_0_) and (*v*_*x*0_, *v*_*z*0_) are the initial position and velocity of the electron.

Equations (5) and (6) show that the electron is accelerated in the *x* direction but it only oscillates in the *z* direction. We can thus consider the transit time *τ*_*transit*_ as the time for the electron to traverse the diffusion region in the *x* direction. Accordingly, from equation (5) we get

7 $${\tau }_{transit}={\tau }_{x}\,\mathrm{ln}(\frac{{D}_{x}+\sqrt{{D}_{x}^{2}+{v}_{x0}^{2}{{\tau }_{x}}^{2}-{x}_{0}^{2}}}{{v}_{x0}{\tau }_{x}+{x}_{0}})$$

where *D*_*x*_ = *x*(*τ*_*transit*_) can be chosen to be at the edge of the simulation box.

Near the X line, we can also reasonably assume that thermal effects can be neglected and the initial in-plane electron velocity is nearly zero. Considering that the electron motion in the *z* direction is oscillatory, for the transit time we only need to follow its motion in the *x* direction. Accordingly, equation (7) becomes

8 $${\tau }_{transit}={\tau }_{x}\,\mathrm{ln}(\frac{{D}_{x}+\sqrt{{D}_{x}^{2}-{x}_{0}^{2}}}{{x}_{0}})$$

Since the initial position *x*_0_ of an electron can be anywhere inside the diffusion region, the mean transit time is

9 $${\bar{\tau }}_{transit}=\frac{1}{{D}_{x}}{\int }_{0}^{{D}_{x}}{\tau }_{transit}d{x}_{0}=\frac{\pi }{2}{\tau }_{x}$$

the result of ${\bar{\tau }}_{transit}$ is independent of *D*_*x*_. So that the effective collisionless resistivity is

10 $${\eta }_{eff}=\frac{2}{\pi }\sqrt{|\frac{{m}_{e}{v}_{y}{B}_{z}}{{e}^{3}{n}^{2}{L}_{x}}|}$$

In deriving the electron transit time, we have made the reasonable but unsubstantiated assumption on the existence of a collisionless, or effective, resistivity that imitates the function of the classical collisional resistivity in the fluid description of the plasma. For verification, we next carry out full PIC simulations of the FMR in collisionless plasma.

## Particle-in-Cell simulation

We have performed 2.5D full PIC simulations for plasma particle motion in the diffusion region by assuming ∂_*y*_ = 0. For simplicity, we use the charge-conservation scheme (CCS) instead of solving the Poisson equation, and the finite difference time domain (FDTD) method to solve the other Maxwell’s equations. The equations used in the PIC simulations are

11 $$\nabla \times {\boldsymbol{E}}=-\frac{\partial {\boldsymbol{B}}}{\partial t}$$

12 $$\nabla \times {\boldsymbol{B}}={\mu }_{0}{\varepsilon }_{0}\frac{\partial {\boldsymbol{E}}}{\partial t}+{\mu }_{0}{\boldsymbol{J}}$$

13 $$\frac{d{{\boldsymbol{p}}}_{j}}{dt}={q}_{j}({\boldsymbol{E}}+{{\boldsymbol{v}}}_{j}\times {\boldsymbol{B}})$$

where *c* is the light speed, ***v***_*j*_ and ***p***_*j*_ = *m*_*j*_***v***_*j*_ are the particle velocity and momentum, respectively. The variables are normalized as follows: ***x***/*d*_*i*0_→***x***, (***V***_*j*_, ***v***_*j*_)/*v*_*Ai*0_→(***V***_*j*_, ***v***_*j*_), *ω*_*ci*0_*t*→*t*, ***B***/*B*_0_→***B***, ***E***/*E*_0_→***E***, ***J***/*J*_0_→***J***, *n*/*n*_0_→*n*, ***p***_*j*_/*m*_*i*_*v*_*Ai*0_→***p***_*j*_, where ${d}_{i0}=c/{\omega }_{pi0}=c/\sqrt{{n}_{0}{e}^{2}/{\mu }_{0}{m}_{i}}$, ${v}_{Ai0}={B}_{0}/\sqrt{{\mu }_{0}{n}_{i0}{m}_{i}}$, *ω*_*ci*0_ = *eB*_0_/*m*_*i*_, *E*_0_ = *v*_*Ai*0_*B*_0_, and *J*_0_ = *n*_0_*ev*_*Ai*0_.

For the PIC simulations, we set ${v}_{Ai0}/c=0.05$, the ion-to-electron mass ratio *μ* = *m*_*i*_/*m*_*e*_ is from 25 to 400, and the initial ion-to-electron temperature ratio is *T*_*i*_/*T*_*e*_ = 5. Our simulation domain is −*D*_*x*_/2≤ *x* ≤*D*_*x*_/2, −*D*_*z*_/2 ≤ *z* ≤*D*_*z*_/2, where *D*_*x*_ = 12.8*d*_*i*0_, *D*_*z*_ = 6.4*d*_*i*0_, *dx* = *dz* = 0.01*d*_*i*0_ and the time step is *ω*_*ci*0_Δ*t* = 0.0002. Periodic and closed boundary conditions are adopted in the *x* and *z* directions, respectively. Nearly 82 million simulation particles for each species are used.

We use the Harris equilibrium as the initial configuration. The initial magnetic field is given by

14 $${B}_{x}={B}_{0}\,\tanh (z/{b}_{0}),{B}_{z}={B}_{y}=0$$

and the initial density profile is

15 $$n={n}_{0}\,{\cosh }^{-2}(z/{b}_{0})+{n}_{b}$$

where *B*_0_ = 1.0, *b*_0_ = 0.5, *n*_0_ = 1.0, *n*_*b*_ = 0.2, and *b*_0_ is the width of the current sheet with the current intensity given by

16 $${J}_{y}=({B}_{0}/{b}_{0}){\cosh }^{-2}(z/{b}_{0})$$

In the simulation, the reconnection process is initiated by a small perturbation of the magnetic field.

Pressure balance yields

17 $$P+{B}^{2}/2=(1+\beta ){B}_{0}^{2}/2$$

where *P* and *B* are the local thermal pressure and magnetic field, *β* = *P*/(*B*^2^/2), and *P* is normalized by ${B}_{0}^{2}/2{\mu }_{0}$. Here we set *β* = 0.2.

## Comparison of the analytical and simulated resistivities

We first examine the *y* component of the velocity *v*_*y*_ of electrons entering and leaving the electron diffusion region. For ion-to-electron mass ratio *μ* = 400, during the peak reconnection period (from *t* = 16 to 17) we found that the average change of *v*_*y*_ is less than 10%. That is, *v*_*y*_ is indeed roughly constant, as assumed in the evaluation of (5) and (6).

**Figure 2:**
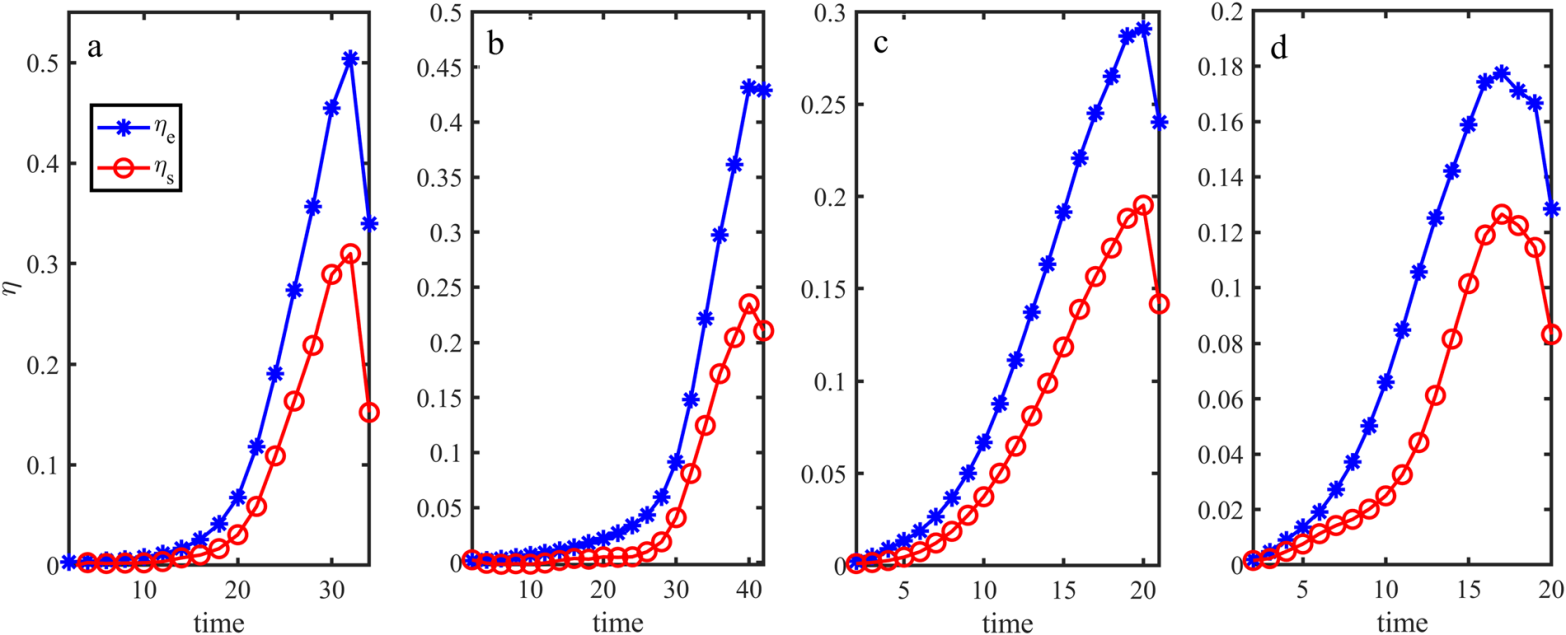
Evolution of the effective resistivity *η*_*eff*_ (blue curve with stars) and the collisionless resistivity *η*_*s*_ (red curve with circles) obtained from the PIC simulations. The panels a to d are for *μ* = 25, 100, 256, and 400, respectively. Note that the quantitative discrepancy decreases as the the mass ratio becomes more and more realistic.

Next we compare the resistivities from our analytical model and the PIC simulation. Figure 2 shows the evolution of the resistivities in the electron diffusion region. In the analytical formula for *η*_*eff*_, the electron number density *n*, velocity *v*_*y*_, *B*_*z*_, and *L*_*x*_ have been replaced by, as calculated from the PIC simulation results, the average electron number density $\bar{n}$ and electron velocity ${\bar{v}}_{y}$ in the electron diffusion region (of size of *d*_*e*_), the maximum *B*_*z*_, and the characteristic length *L*_*x*_ of *B*_*z*_ in the X-point region, respectively. On the other hand, the resistivity from the PIC simulation is calculated directly from the relation *η*_*s*_ = *E*_*y*_/*J*_*y*_ by substituting the measured values of the average electric field intensity ${\bar{E}}_{y}$ and sheet-current density $\bar{J}$. We see that our effective resistivity agrees quite well with the collisionless resistivity obtained from the PIC simulations. In particular, in the fast reconnection phase both resistivities increase rapidly and in a similar manner, thereby verifying the scaling of the field parameters in our model effective resistivity. We can also see that the peak value of the resistivity decreases with increase of the mass ratio *μ*, and the analytical and simulation results approach each other.

## Discussion and Summary

In collisional plasma, the characteristic thickness of the current sheet taking into account magnetic field diffusion is^18,19^ ${{\rm{\Delta }}}_{SP}=\sqrt{{\eta }_{spz}L/{\mu }_{0}{v}_{A}}$, where *L* is the plasma size, *v*_*A*_ is the Alfven speed, and *η*_*spz*_ is the Spitzer resistivity (based on Coulomb collisions). For the parameters of the Earth’s magnetopause and magnetotail^20^, the half-thickness of the thinnest current sheet during the nonlinear stage of magnetic reconnection can be of the order 10 *m* that is four to five orders of magnitude smaller than that obtained from the satellite data during magnetic reconnection: namely, ~100 *km* in the magnetopause^21,22^ and ~1000 *km* in the magnetotail^23,24^. Such a huge descrepancy suggests that Coulomb collisions are not the dominant dissipation mechanism for MR in the magnetopause and magnetail.

The effective resistivity in our model can be rewritten in the form of a power law:

18 $${\eta }_{eff}=2{\pi }^{-1}{\alpha }^{0.5}{\mu }_{0}^{-0.5}{m}_{e}^{0.5}{e}^{-2}{L}_{z}^{-1}{n}^{-1.5}{B}_{0},$$

where we have used the relations $$ne{v}_{y}={\mu }_{0}^{-1}{\partial }_{z}{B}_{x}$$$$ \sim {\mu }_{0}^{-1}{B}_{0}/{L}_{z}$$, as well as the relation $${\partial }_{x}{B}_{z} \sim \alpha {\partial }_{z}{B}_{x}$$ between the local magnetic field components near the X line. The parameter *α* is thus the ratio of the characteristic lengths of *B*_*x*_ and *B*_*z*_. Simulations have shown that in the MR configuration *α* increases as reconnected magnetic field *B*_*z*_ increases with time in the diffusion region and *α* is of order 0.1 when MR gets into the nonlinear stage. In the magnetotail^20^ on Earth’s nightside, the electron density is *n*_*e*_(*n*_*i*_) ≈ 0.3*cm*^−3^, the electron temperature is *T*_*e*_ ≈ 600*eV*, the magnetic field is *B*_0_ ≈ 2 × 10^−8^*T*, and plasma size *L* ≈ 100*R*_*E*_, the current sheet thickness is about Δ_*SP*_ ≈ 0.15*R*_*E*_ ≈ 953*km* in the nonlinear phase of magnetic reconection with *α* = 0.1. On the other hand, for the magnetopause^20^ on the Earth’s dayside, with electron density *n*_*e*_(*n*_*i*_) ≈ 10*cm*^−3^, electron temperature *T*_*e*_ ≈ 300*eV*, magnetic field *B*_0_≈5 × 10^−8^*T*, and plasmas size *L* ≈ 10*R*_*E*_, the current sheet thickness is Δ_*SP*_ ≈ 0.021*R*_*E*_ ≈ 137*km*. Thus, for both the magnetopause and magnetotail, the current sheet thicknesses as predicted by our model are in good agreement with that from the satellite observations. Moreover, one can easily show that for the parameters of the experimental device MRX^20^, our model yields Δ_*SP*_ ≈ 3.38 *cm*, which is in good agreement with that from the direct laboratory measurement.

**Figure 3:**
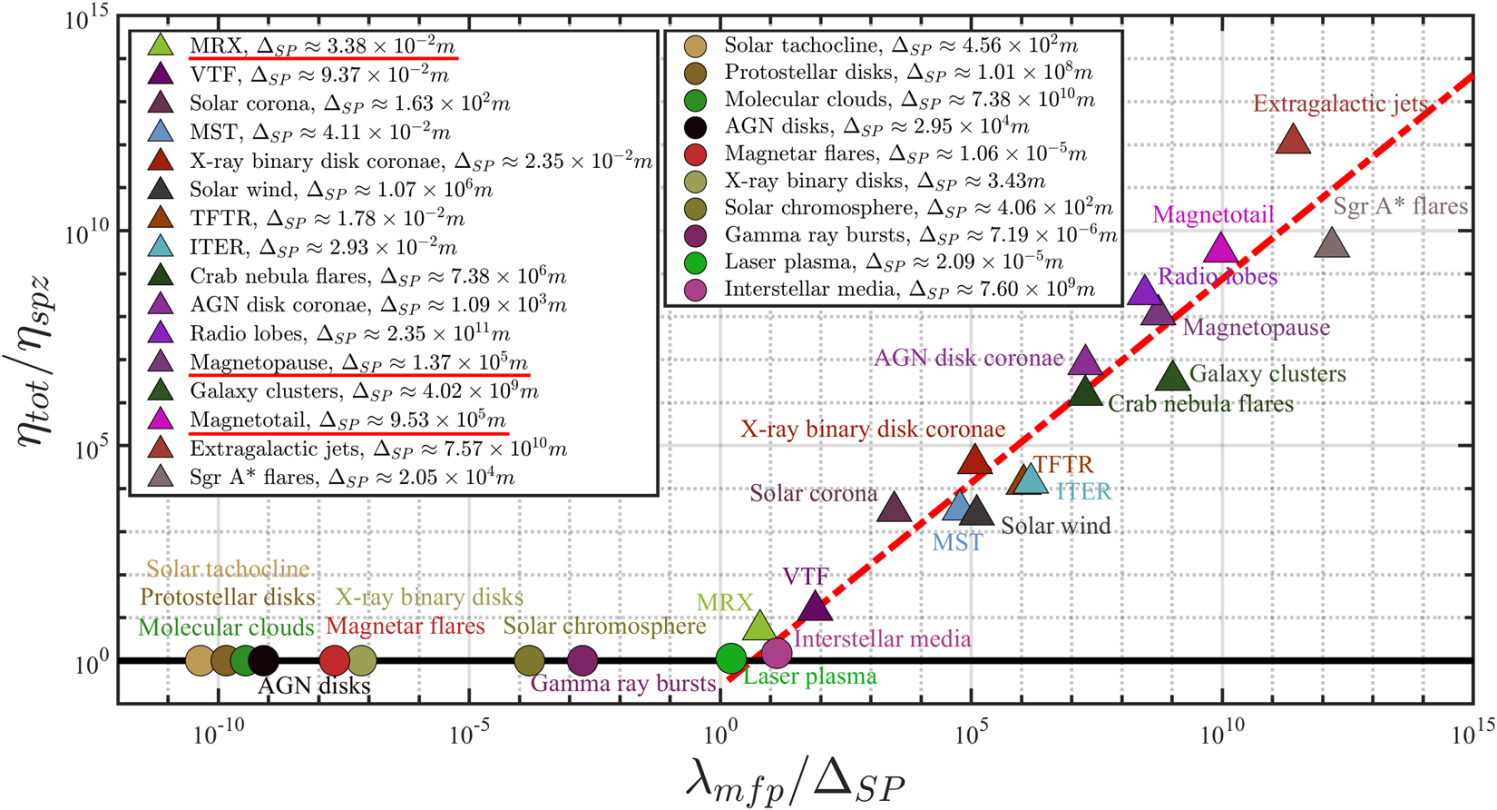
Plot of the normalized total resistivity *η*_*tot*_/*η*_*spz*_ for space and laboratory plasmas exhibiting MR versus the normalized mean-free-path *λ*_*mfp*_/Δ_*SP*_. Here, the thickness of the current sheet according to the Sweet-Parker model is ${{\rm{\Delta }}}_{SP}=2{L}_{z} \sim 2L/{S}_{L}^{1/2},$ where *S*_*L*_ = *μ*_0_*Lv*_*A*_/*η*_*tot*_ is the Lundquist number, or the ratio of the resistive to Alfvèn times, and *L* is the overall plasma size. Note that the normalization parameters *η*_*spz*_ and Δ_*SP*_ are different for different data points. The solid circles and triangles are for collisional (*λ*_*mfp*_/Δ_*SP*_ <1) and collisionless (*λ*_*mfp*_/Δ_*SP*_ >1) plasmas, respectively. The corresponding current sheet thicknesses Δ_*SP*_ based on our effective resistivity are given in the insets. The red dashed line is the best fit for the data points in the collisionless plasma regime (*λ*_*mfp*_/Δ_*SP*_ > 1).

It is of interest to make a broader comparison of the results from our model with that from existing data on space and laboratory plasmas where MR is observed or expected to exist. Since classical collisions can be important or relevant in some of the environments^20^, it is useful to introduce the total resistivity *η*_*tot*_=*η*_*spz*_+*η*_*eff*_. Figure 3 shows the plot of *η*_*tot*_ normalized by the Spitzer resistivity *η*_*spz*_ versus the mean-free-path *λ*_*mfp*_ normalized by the Sweet-Parker current sheet thickness Δ_*SP*_ = 2*L*_*z*_. From Fig. 3, it is clearly shown that all data points are distributed near the best-fitting line. Since ${\eta }_{spz}={m}_{e}{v}_{the}/n{e}^{2}{\tau }_{mft}{v}_{the}={m}_{e}\sqrt{3k{T}_{e}/{m}_{e}}/n{e}^{2}{\lambda }_{mfp}$, we have the power-law scaling *η*_*eff*_/*η*_*spz*_ = [4*π*^−1^*α*^0.5^(3*μ*_0_*kT*_*e*_*n*_*e*_)^−0.5^*B*_0_]*λ*_*mfp*_/Δ_*SP*_ = *Cλ*_*mfp*_/Δ_*SP*_, should correspond to the slope of the data points in the figure. For *α* = 0.1 in the nonlinear stage of magnetic reconnection, we find that *C* is in the range 3 × 10^−3^ to 4 for collisionless (*λ*_*mfp*_/Δ_*SP*_ > 1) plasmas. The slope of the best-fit (red dashed) line in Fig. 3 is *C* = 0.1, which is in the middle of its range. We note that the data points only slightly deviate from the best-fit line, and can be attributed to uncertainties in the obervational data. The somewhat larger deviation for the data points from the magnetic-confinement-fusion devices ITER and TFTR can be attributed to the strong guiding magnetic field. It is well known that a guide field can suppress magnetic reconnection or reduce the effective resistivity, which is consistent with the fact that the ITER and TFTR data points are located below the best-fit line. The results here suggest that pitch angle scattering of electrons due to bending magnetic field lines, which is the basic assumption of our model, may be responsible for fast MR in collisionless plasmas.

The effective resistivity in our 2D model is mainly a result of diversion of electrons in the electron diffusion region, which has very small spatial scale. Three-dimensional (3D) effects are ignorable if the spatial scale of the magnetic field in the third direction is larger than the electron inertia length. This condition is usually valid for space and laboratory plasmas, which explains why the effective resistivity from our model agrees very well with that of a large number of environments, namely from intergalactic and interstellar to solar and terrestrial to laboratory fusion plasmas. The reconnection rate in general depends on the ratio between the thickness and length of the diffusion region, or the current sheet. The thickness is usually determined by dissipation, or resistivity, of the system. Therefore, for given resistivity, the reconnection rate can increase with decrease of the current sheet length, say by an external driving force. For example, in tokamak plasmas, the slower tearing modes correspond to spontaneous MR and the sawtooth oscillations correspond to FMR driven by internal kink instabilities.

In summary, for understanding FMR we have introduced an effective resistivity that contains no free parameters. The effective resistivity is based on self-consistent scattering or acceleration of electrons by bending of magnetic field lines, and it agrees in magnitude with that of a large number of environments where MR is observed or suspected. It can also be readily adapted in existing collisional-fluid simulation codes for investigating collisionless FMR. The present work can also serve as a guide for a formal first-principles derivation of such an effective resistivity. Finally, it may be of interest to point out that our results are clearly also applicable to very small scale and very fast MR in the absence of ion dynamics. In fact, such novel ultrafast (45 millisecond) MR phenomena have been recently reported to be occuring within the entangled magnetic fields in the Earth’s turbulent magnetosheath.^25^
